# Different aspects of failing to recover from proactive semantic interference predicts rate of progression from amnestic mild cognitive impairment to dementia

**DOI:** 10.3389/fnagi.2024.1336008

**Published:** 2024-01-31

**Authors:** Rosie E. Curiel Cid, Elizabeth A. Crocco, Ranjan Duara, David Vaillancourt, Breton Asken, Melissa J. Armstrong, Malek Adjouadi, Mike Georgiou, Michael Marsiske, Wei-in Wang, Monica Rosselli, William W. Barker, Alexandra Ortega, Diana Hincapie, Liz Gallardo, Feras Alkharboush, Steven DeKosky, Glenn Smith, David A. Loewenstein

**Affiliations:** ^1^Florida Alzheimer’s Disease Research Center, Miami, FL, United States; ^2^Center for Cognitive Neuroscience and Aging, Department of Psychiatry and Behavioral Sciences, University of Miami Miller School of Medicine, Miami, FL, United States; ^3^Department of Neurology and The Center for Translational Research in Neurodegenerative Disease, University of Florida, Gainesville, FL, United States; ^4^Department of Applied Physiology and Kinesiology, Gainesville, FL, United States; ^5^University of Florida College of Medicine, Gainesville, FL, United States; ^6^Department of Clinical and Health Psychology, University of Florida, Miami, FL, United States; ^7^Department of Psychology, Florida Atlantic University, Boca Raton, FL, United States; ^8^Wien Center for Alzheimer’s Disease and Memory Disorders, Mount Sinai Medical Center, Miami, FL, United States; ^9^Department of Neurology and McKnight Brain Institute, University of Florida, Gainesville, FL, United States; ^10^Center for Advanced Technology and Education, Florida International University, Miami, FL, United States

**Keywords:** mild cognitive impairment, Alzheimer’s dementia progression, proactive semantic interference, LASSI-L, amyloid imaging, structural MRI

## Abstract

**Introduction:**

This study investigated the role of proactive semantic interference (frPSI) in predicting the progression of amnestic Mild Cognitive Impairment (aMCI) to dementia, taking into account various cognitive and biological factors.

**Methods:**

The research involved 89 older adults with aMCI who underwent baseline assessments, including amyloid PET and MRI scans, and were followed longitudinally over a period ranging from 12 to 55 months (average 26.05 months).

**Results:**

The findings revealed that more than 30% of the participants diagnosed with aMCI progressed to dementia during the observation period. Using Cox Proportional Hazards modeling and adjusting for demographic factors, global cognitive function, hippocampal volume, and amyloid positivity, two distinct aspects of frPSI were identified as significant predictors of a faster decline to dementia. These aspects were fewer correct responses on a frPSI trial and a higher number of semantic intrusion errors on the same trial, with 29.5% and 31.6 % increases in the likelihood of more rapid progression to dementia, respectively.

**Discussion:**

These findings after adjustment for demographic and biological markers of Alzheimer’s Disease, suggest that assessing frPSI may offer valuable insights into the risk of dementia progression in individuals with aMCI.

## 1 Introduction

It has been long recognized that interference effects on competing words, objects, and other stimuli may have profound effects on memory processes, and rank among the most potent contributors to observed short-term memory deficits (Atkins et al., 2011). Two classic interference paradigms are commonly referred to in the literature: proactive interference (PI) in which old learning interferes with new learning, and retroactive interference (RI), in which new learning interferes with old learning (Keppel and Underwood, 1962; Keppel et al., 1971). Phonological, physical, contextual, and semantic similarities of to-be-remembered targets can enhance interference effects (Loewenstein et al., 2004; Atkins et al., 2011; Crocco et al., 2014). The failure to recover from proactive semantic interference (frPSI) measures the persistent inability to learn new, semantically competing stimuli, despite multiple opportunities to do so. Recent findings from several international laboratories have shown that among older adults at risk for cognitive decline, frPSI persists when competing word lists are used. It is important to note that this paradigm is different from the release of proactive interference (i.e., reducing interference by switching from remembering names to remembering dates). Indeed, frPSI is among the earliest detectable symptoms of Alzheimer’s disease and occurs years before the onset of frank cognitive impairment or dementia (Loewenstein et al., 2017a; Sánchez et al., 2017; Matias-Guiu et al., 2018).

**Figure 1:**
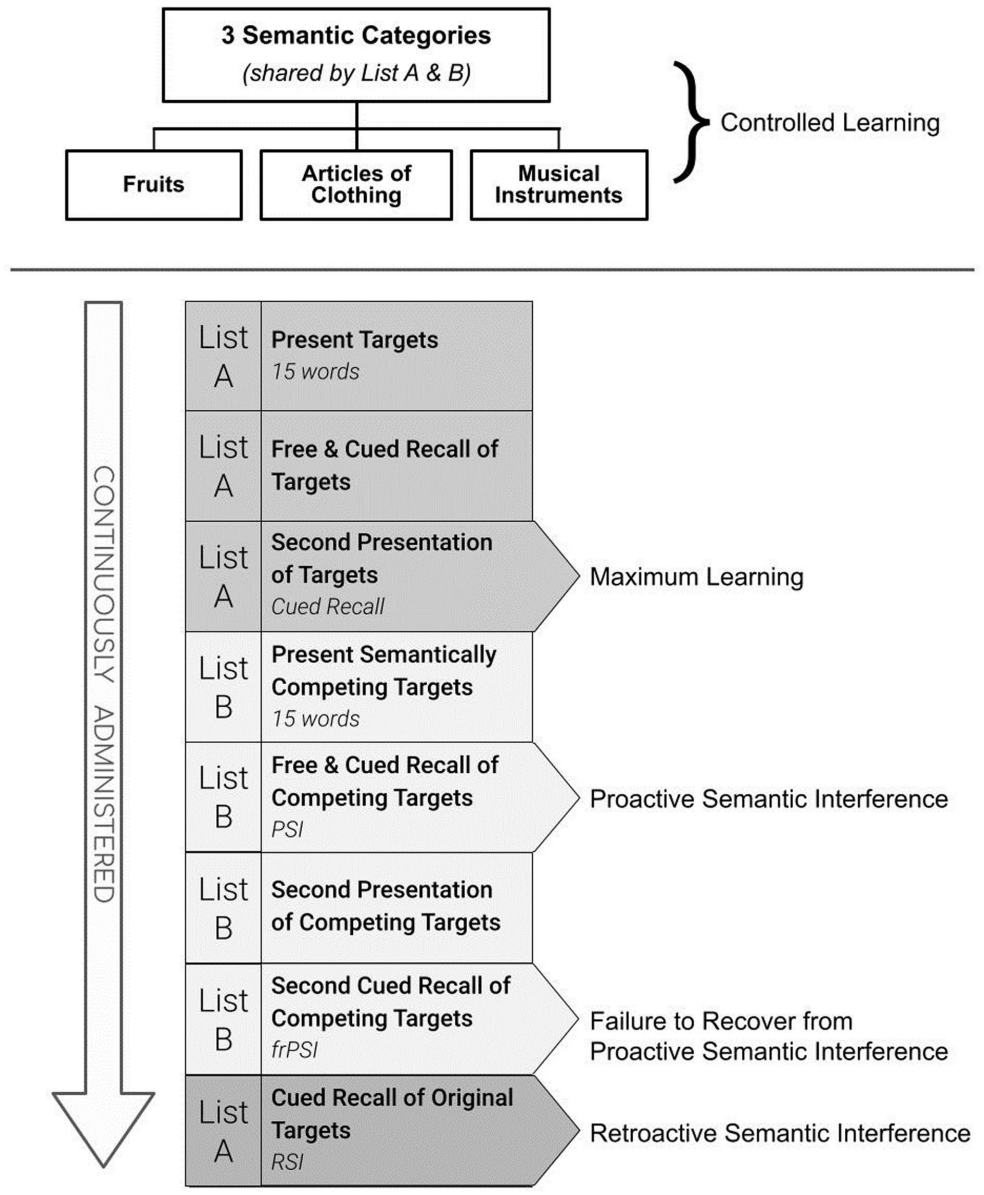
Different aspects of learning and memory assessment using the LASSI-L.

These replicated findings have stimulated interest in unraveling different aspects of this newly identified cognitive impairment. FrPSI is uniquely measured by the Loewenstein and Acevedo Scales for Semantic Interference and Learning (LASSI-L) (Curiel et al., 2013). [See Figure 1]. During administration, the examinee is instructed to remember a list of 15 common words where each word belongs to one of three distinct semantic categories for two learning trials. The words are presented using a controlled learning strategy where category cues are given both at the time of initial learning and during recall trials (List A). A second competing list of words (List B) is then presented. List B is also comprised of 15 words that share the identical semantic categories as List A. Unlike traditional memory assessments, the LASSI-L paradigm incorporates a second-word list (List B) that semantically competes with the words they recently learned (List A). This has the potential to elicit a considerable amount of PSI as measured by the reduced number of correct responses when the examinee is given the semantic cue and asked to remember the competing words (Cued B1 Recall).

Detecting frPSI deficits involves the additional presentation and cued recall of List B (Cued B2). Thus, two ways to examine the frPSI deficits include measuring total correct responses on the second cued recall trial of List B (Cued B2 Recall), as well as the semantic intrusion errors that occur during the Cued B2 recall trial. When compared to older adults who are cognitively unimpaired, persons diagnosed with amnestic mild cognitive impairment (aMCI) fail to recover from PSI (Loewenstein et al., 2018a; Cid and Loewenstein, 2022). In persons with aMCI, this failure to recover from PSI manifests despite multiple learning trials, and even after adjusting for maximum learning performance measured by the total number of words learned after the second cued recall of the first list (A2 Cued Recall). Similarly, compared to demographically similar controls, those classified as Preclinical Mild Cognitive Impairment (PreMCI) who lacked the objective cognitive decline required for a formal diagnosis of MCI but experienced subjective memory complaints and evidence of memory decline by clinical evaluation, showed this pattern (Curiel et al., 2018a). This same phenomenon has been observed using different category cues with as many as 18 targets presented for two competing word lists, even over three cued recall trials, suggesting that the failure to recover from PSI occurs despite optimizing the opportunity to learn through repeated exposure (Loewenstein et al., 2021).

The most common practice across neuropsychological tests assessing word-list learning is to examine the number of correct responses produced on the word list. However, we have found that offering category cues to organize learning and elicit recall of semantically competing lists, by its nature, elicits a number of semantic intrusion errors in persons who are at risk for AD and AD-related disorders. This occurs even after examinees are provided with multiple learning trials for both competing lists in a standardized fashion. The vast number of these errors, reflecting intrusions of target words from the first list, is thought to be a different manifestation of frPSI during the second cued recall trial for List B. According to Torres et al. (2019), 90 percent of the semantic intrusion errors that occur when recalling List B words are intruded from List A.

This has been conceptualized as a failure to both monitor and inhibit competing semantic responses (Cid and Loewenstein, 2022). Intrusion errors can be important early markers of subtle brain dysfunction (Thomas et al., 2018) particularly AD-related memory changes. Greater numbers of semantic intrusions differentiate individuals with aMCI who are amyloid PET positive from aMCI who are amyloid PET negative (Loewenstein et al., 2018b) while the other neuropsychological deficits studied did not. Semantic intrusion errors have also differentiated amyloid PET-positive aMCI from other neurological and neuropsychiatric conditions with MCI due to non-AD conditions that were amyloid PET-negative (Kitaigorodsky et al., 2021). Semantic intrusion errors that occur on measures of failure to recover from PSI have also been associated with corticolimbic disconnection in asymptomatic middle-aged children of a parent with AD (Sánchez et al., 2017).

Two distinct aspects of frPSI are as follows: (a) the continued suppression of correct responses despite a second learning trial because of putative interference from competing items from the first list of words (List A); and (b) the inability to inhibit competing responses from the first list (semantic intrusions), despite multiple opportunities to do so (Torres et al., 2019; Loewenstein et al., 2021). These two aspects of frPSI may be measured independently and may be important in distinguishing those with aMCI who are amyloid positive from those who are amyloid negative (Loewenstein et al., 2018b; Kitaigorodsky et al., 2021). This has the potential to improve screening for AD, particularly in settings where biomarkers are not readily available or to refer for more advanced biomarker assessment for diagnostic clarification, prognostication, and/or inclusion in emerging AD clinical trials.

Despite the intriguing possibility that there may be different manifestations of frPSI, studies to date have been cross-sectional and have not examined the prognostic implications of different aspects of frPSI. This has practical as well as theoretical implications since the likelihood and/or rate of progression from aMCI to dementia impacts patients and their families. Further, it is not known whether, or to what extent, biological variables such as PET amyloid status, hippocampal volume, sex, or other demographic factors influence the likelihood that correct responses and number of semantic intrusions on frPSI predict the rate of progression of aMCI to dementia when all variables undergo simultaneous adjustment in a robust multivariate model. In the present study, Cox Proportional Hazard Modeling was employed to determine if one or both LASSI-L measures of frPSI were predictive of the rate of progression over time from aMCI to dementia. We hypothesized that both correct responses and semantic intrusion errors represented unique aspects of frPSI which could both independently predict faster rates of progression to dementia after adjusting for important covariates in statistical models.

## 2 Methods

### 2.1 Baseline diagnostic criteria

Participants were enrolled in the 1Florida Alzheimer’s Disease Research Center. Baseline diagnoses were established in the following manner. An experienced bilingual clinician (fluent in Spanish and English), blind to neuropsychological test results, administered a standard clinical assessment protocol, which included the Clinical Dementia Rating Scale (CDR) (Morris, 1991), and the Mini-Mental State Examination (MMSE) (Folstein et al., 1975) to assess memory and other clinical and cognitive complaints. All participants were community-dwellers, independent in their activities of daily living, had knowledgeable collateral informants, and did not meet DSM-V criteria for Major Neurocognitive Disorder, an active Mood or Psychotic Disorder, or any other neuropsychiatric disorder (American Psychiatric Association DS and Association AP, 2013). In cases where there was evidence of memory decline by history and/or clinical examination, the clinician scored the Global CDR as 0.5 and assigned a diagnosis of probable amnestic MCI (aMCI), pending the results of formal neuropsychological testing.

Subsequently, independent of the clinical examination, a bilingual-trained psychometrician administered a standard neuropsychological battery including the LASSI-L. To avoid circular reasoning (aka criterion contamination), the LASSI-L was not part of the cognitive diagnostic process. The neuropsychological battery used to classify older adults into groups included the Hopkins Verbal Learning Test-Revised (HVLT-R) (Benedict et al., 1998) immediate and delayed memory, delayed recall on the NACC story passages (Beekly et al., 2007), the Controlled Oral Word Association Test: Category Fluency (Binetti et al., 1995), Block Design subtest of the Wechsler Adult Intelligence Scale, Fourth Edition (WAIS-IV) (Wechsler, 2008), and the Trail Making Test (Parts A and B) (Retain, 1958).

### 2.2 Diagnosis of amnestic mild cognitive impairment

On the basis of the independent clinical interview and performance on the neuropsychological tests, 89 individuals were classified as aMCI. All of these individuals met the following criteria: (a) subjective memory complaints by the participant and/or collateral informant; (b) evidence by clinical evaluation or history of memory and/or other cognitive decline; (c) Global CDR score of 0.5; (d) one or more memory measures Hopkins Verbal Learning Test- Revised (HVLT-R) (Benedict et al., 1998) immediate and delayed memory, delayed recall on the National Alzheimer’s Coordinating Center’s (NACC) story passages (Beekly et al., 2007), below normal limits (i.e., a score below 1.5 SD or more relative to age, education, and language-adjusted normative data).

### 2.3 Longitudinal classification

All individuals with aMCI and full baseline cognitive and neuropsychological evaluation, MRI evaluation, and amyloid PET at baseline were followed approximately annually for a period of 12 to 55 months (mean = 26.05; SD = 11.0). The time-to-event in Hazards Modeling was determined by the number of weeks an individual took to progress from a diagnosis of aMCI with a global CDR of 0.5 to a diagnosis of dementia with a global CDR ≥ 1.0. All of those who progressed to dementia met DSM-5 criteria for a Major Neurocognitive Disorder (American Psychiatric Association DS and Association AP, 2013). For those who did not progress, the number of weeks from their first to last visit, up to 55 months, were recorded. This allowed for correct censoring of data using Cox regression.

### 2.4 Loewenstein–Acevedo scales for semantic interference and learning

It is well established that the time to progression to dementia for aMCI participants is strongly related to (a) the initial severity of cognitive impairment at the time of diagnosis; and (b) the number of cognitive domains impaired (Cid and Loewenstein, 2022). Using the same cognitive tests to help render an initial diagnosis and then proceeding to use these same measures to help determine progression to dementia can introduce potential circularity when these same measures are employed as predictors of outcome. For this reason, the LASSI-L was not used for diagnostic determination in any aspect of this study to avoid potential issues of circularity or potential tautology. The LASSI-L cognitive stress test employs a controlled learning paradigm at acquisition in an effort to maximize the storage of a list of to-be-remembered target words belonging to three distinct semantic categories (fruits, clothing, and musical instruments; Loewenstein et al., 2016). Participants were tested in their preferred language (English versus Spanish) and the LASSI-L has been previously shown to be culturally fair and valid in either language (Matías-Guiu et al., 2017; Curiel Cid et al., 2019). During the administration of the LASSI-L, the examinee was asked to remember a list of 15 common words representing three semantically distinct categories over two learning trials to maximize storage and consolidation (List A Cued Recall). Subsequently, there was the presentation of a second competing list of to-be-remembered words presented in the same manner as the first list. The second list (List B) introduced different target words, but they all represented the identical presented semantic categories, used in List A in order to elicit maximum levels of proactive interference when recalling the second set of items (PSI). Unlike other traditional memory assessment paradigms, the re-administration and subsequent recall of this second list of words measures the individual’s ability to recover from the effects of PSI (frPSI) and is captured by correct responses on Cued B2. Maximum initial learning (Cued A2) and retroactive semantic interference again assessing Cued A2 targets on the LASSI-L after an initial recall was not examined since this has not been found to be particularly useful (Loewenstein et al., 2016, 2021). For the current study, our focus was on different aspects of frPSI, since these aspects of the LASSI-L have had the most predictive utility in many studies nationally and internationally (Curiel Cid et al., 2023). As such, we focused on (a) correct responses and (b) semantic intrusion errors that occurred on the List B2 Cued Recall subscale of the LASSI-L. These measures tap failure to recover from PSI and have previously demonstrated excellent discriminatory power in differentiating aMCI from cognitively unimpaired older adults and have been highly related to neurodegeneration in AD-prone regions (Loewenstein et al., 2016, 2017b). Intrusion errors on these subscales have also been sensitive to the downstream effects of amyloid load (Curiel et al., 2018b; Loewenstein et al., 2018b; Kitaigorodsky et al., 2021) including tau deposition, and neurodegeneration thought to reflect deficits in self-monitoring, and inhibitory control (Loewenstein et al., 2018a; Torres et al., 2019; Cid et al., 2020).

### 2.5 Amyloid PET imaging scans

PET/CT imaging was obtained using a 3D Hoffmann brain phantom to establish a standardized acquisition and reconstruction method. Participants were infused with [18-F] florbetaben 300 MBQ over a 3-min period. Scanning commenced 70–90 min after an infusion duration of 20 min. We scanned all participants on a Siemens Biograph 16 PET/CT scanner operating in 3D mode (55 slices/frame, 3 mm slice thickness 128 ×128 matrix). The PET data were reconstructed into 128 × 128 × 63 (axial) matrices with voxel dimensions of 0.21 × 0.21 × 0.24 cm. Reconstruction was performed using manufacturer-supplied software and included corrections for attenuation, scatter, random coincidences, and dead time. Images for regional analyses were processed using Fourier analysis followed by direct Fourier reconstruction. Images were smoothed with a 3 mm Hann filter. Following reconstruction, image sets were inspected and, if necessary, corrected for inter-frame motion. Images were obtained from the top of the head to the top of the neck and computed tomography (CT) data were employed for initial attenuation correction and image reconstruction in the sagittal, axial, and coronal planes.

The PET/CT scans, including the outline of the skull were co-registered linearly (i.e., trilinear interpolation) with 12 degrees of freedom, onto the volumetric MRI scan using a T1-weighted MP-RAGE image (Lizarraga et al., 2016). Region-of-interest (ROI) boundaries were defined manually using the structural MRI for anatomical reference, and criteria that have been proven to provide highly reproducible outcomes (Desikan et al., 2006). This registration process ensured that the PET/CT image had the same accurate segmentation and parcellation as in the MRI scan. The PET/CT and MR reconstruction was performed using the manufacturer-provided software. Registration between PET/CT and the MR, FSL’s FLIRT (Jenkinson et al., 2002) algorithm was employed. For deriving the ROIs, MR images were segmented using FreeSurfer 6.0 and the derived ROIs were superimposed on the registered PET images to derive the regional values. Average activity was calculated in the ROIs corresponding to cerebellar gray matter and cerebral cortical regions. A composite Standardized Uptake Value Ratio (SUVR) was calculated by the ratio of the mean volume weighted SUVR of 5 bilateral cortical regions (frontal, temporal, parietal, anterior and posterior cingulate cortex), to the cerebellar gray matter (Rowe et al., 2008). While over 80 percent of our amyloid scans utilized florbetaben as the primary tracer, a small minority of our subjects had florbetapir scans. The Centiloid method which has been widely used to create a common metric by which total amyloid uptake can be placed on the same scale for different amyloid tracers (Jack et al., 2016; Rowe et al., 2017). Using normalization to the whole brain cerebellum, for florbetaben, the Centiloid formula is [(SUVR X 153.4) -154.9] and for florbetapir, [(SUVR X 183) -177]. This created a Centiloid score for each participant (Klunk et al., 2015).

### 2.6 Visual ratings of amyloid PET scans

All amyloid PET scans were interpreted using a methodology similar to that described by Cid et al. (2020), by an experienced neuroradiologist, Dr. Ranjan Duara, who was blind to the cognitive and clinical diagnoses. Using the same methodology, the aforementioned authors reported an interrater reliability of 98% for amyloid visual reads between Dr. Ranjan Duara and an independent rater. A final dichotomous amyloid positive (A+) versus amyloid negative (A-) diagnosis was rendered. Visual amyloid reads are considered the gold standard in the field (Duara et al., 2019).

### 2.7 Assessment of neurodegeneration using MRI

A Siemens Skyra 3 T MRI scanner (Siemens Medical Solutions, Erlangen, Germany) at Mount Sinai Medical Center, Miami Beach, Florida. The 1-h MRI acquisition was 1-h. The 3D T1 weighted volumetric magnetization-prepared rapid gradient-echo sequence (MP-RAGE) consisted of 176 slices at slice thickness = 1 mm isotropic, FOV = 256 × 256, TR = 3.0 s, TE = 1.4 s, and flip angle = 9 degrees The whole hippocampal volume was calculated using FreeSurfer 6.0 software.^1^

### 2.8 Statistical analyses

Group means for converters versus non converters were assessed by one-way analyses of variance (ANOVA). In cross-sectional analyses, statistical adjustments were made for variables such as initial MMSE scores between progressors and non-progressors and were analyzed using analyses of covariance (ANCOVA). Differences in proportions were examined by chi-square analysis with Yates Correction for discontinuity. Pearson correlation coefficients were used to construct covariance matrices between LASSI- L-L frPSI and other neuropsychological measures. Longitudinal analyses were conducted using Cox Proportional Hazards modeling with simultaneous entry so that each predictor variables or covariate was adjusted for in the model. Covariates included age, sex, level of education, Hispanic ethnicity, MMSE total score, amyloid PET status (visual read), hippocampal volume, and interval of last follow-up. All dichotomous variables were coded 1 or 0. value of *p*s for statistical significance were set at *p* < 0.05.

## 3 Results

aMCI progressors as a whole did not statistically differ from aMCI non-progressors on demographic variables such as level of education, sex, Hispanic ethnicity, or interval of last follow-up (Table 1). aMCI progressors were slightly older (non-significant): 74.4 years (SD = 8.5) versus 71.2 years (SD = 6.5); [*F* (1,88) = 3.66; *p* = 0.059]. In addition, aMCI progressors had lower baseline MMSE score relative to non-progressors [26.00 (SD = 1.8) versus 28.15 (SD = 1.6)], [*F* 1,88 = 31.16; *p* < 0.001]. Non-progressors had greater hippocampal volumes at baseline than progressors, and a lower percentage of amyloid positivity. aMCI progressors had fewer correct scores on Cued B2 recall subject to frPSI: 7.04 (SD 3.4) versus 9.76 (SD = 2.5); [*F* (1,88) = 22.93; *p* < 0.001] and higher frPSI intrusion scores: 5.26 (SD = 3.4) versus 2.35 (SD = 2.0); [*F* (1,88) = 24.59; *p* < 0.001]. aMCI progressors continued to have significantly lower frPSI correct scores and more semantic intrusion errors on frPSI (*p* < 0.001), after adjusting for baseline MMSE scores.

**Table 1:** Demographic variables for progressor versus non-progressor aMCI groups.

	aMCI Non-Progressors (*n* = 62)	aMCI Progressors (*n* = 27)	*F* value or *X*^2^	Value of *p*	*P*-Value Adjusting for MMSE
Age (range 56–98)	71.23 (SD = 6.5)	74.37 (SD = 8.5)	3.66	0.059	NA
Education (range 6–22)	15.26 (SD = 3.2)	14.89 (SD = 3.6)	0.227	0.635	NA
MMSE (range 22–30)	28.15 (SD = 1.6)	26.00 (SD = 1.8)	31.16	<0.001	NA
Sex (%female)	44.6%	54.4%	0.035	0.832	NA
Hispanic/Latino Percentage	58.4%	41.%	0.413	0.520	NA
Maximum Testing Interval (range12-55)	27.06 (SD = 11.3)	24.07 (SD = 10.2)	1.40	0.241	NA
Percentage Read as Amyloid Positive	45.5%	61.5%	4.17	0.041	NA
Percent HPC Atrophy (range = 0.00296–0.00763)	0.0048 (SD = 0.0005)	0.0049 (SD = 0.0006)	10.24	**<0.002**	NA
Cued B2 Recall (frPSI) (range 3–15)	9.76 (SD = 2.5)	7.04 (SD = 2.4)	22.93	**<0.001**	**<0.001**
Cued B2 Intrusions (range 0–13)	2.35 (SD = 2.0)	5.26 (SD = 3.4)	24.59	**<0.001**	**<0.001**

Bolded items denote statistical significance at *p* < 0.05.

A correlation matrix between LASSI-L frPSI measures and other neuropsychological variables. While participants had all memory measures, only two-thirds of participants had not memory measures available. As expected LASSI-L Cued B2 correct recall was highly associated with other neuropsychological measures of recall such as the HVLT-R immediate Recall (*r* = 0.632; *p* < 0.001), NACC passage delayed recall (*r*=0. 534) and language measures such as Category Fluency (*r* = 0.489; *p* < 0.001). No statistically significant correlation coefficients were observed for Trails B; (*r* = -239; *p* = 0.074):or the Mint naming Test (*r* = .0.199; *p* = 0.133). In contrast, LASSI-L Cued B2 recall and Cued B2 frPSI semantic intrusion errors was (*r* = −0.311; *p* = 0.003), a modest association. The correlation between Cued B2 frPSI semantic intrusion errors and HVLT-R immediate Recall was (*r* = -303; *p* = 0.003) and NACC passage delayed recall (*r* = .-3.43; *p* < 0.001) and language measures such as Category Fluency (*r* = −0.419): *p* < 0.001. A statistically significant correlation coefficients were observed for Trails B (*r* = .0.388; *p* = 0.003) but not the Mint naming Test (*r* = 0.247; *p* = 0.061) (See Table 2).

Among those with aMCI, 27 of the 89 participants or 30.3% progressed to dementia. We performed Cox Proportional Hazards Modeling with demographic variables, MMSE scores, amyloid status, and hippocampal volume all simultaneously entered and adjusted for in the model. For the omnibus test of model coefficients, a Chi-square of 47.05 (df = 9) was statistically significant at *p* = 0.001. With simultaneous entry for covariates, Table 3 demonstrates that demographic variables such as age, educational attainment and sex did not enter into the model. Those of Hispanic Ethnicity tended to decline at a faster rate [*B* = 0.1.110; (SE = 1.56); Wald = 3.950; *p* = 047] but the range 95% confidence interval was extremely large.

**Table 2:** Association between LASSI-L frPSI variables and traditional neuropsychological measures.

	Cued B2 Correct Score subject to frPSI	Cued B2 Semantic Intrusions subject to frPSI	HVLT-R Total Recall	Delayed NACC Passage	NACC Category Fluency	Trails B	MINT Naming
Cued B2 Correct Score subject to frPSI	NA	−0.315 (*p* < 0.003)	0.632 (*p* < 0.001)	0.534 (*p* < 0.001)	0.489 (*p* = 0.001)	−0.239 (*p* = 0.074)	0.199 (*p* = 0.133)
Cued B2 Semantic Intrusions subject to frPSI	−0.311 (*p* < 0.003)	NA	−0.303 (*p* <=003)	−0.343 (*p* < 0.001)	−0.419 (*p* < 0.001)	0.388 (*p* = 0.003)	−247 (*p* = 0.061)

Only 2/3 of the sample had non-memory measures.

**Table 3:** Proportional hazards model examining rate of progression to dementia among persons with an initial diagnosis of aMCI on LASSI-L measures susceptible to different aspects of frPSI, amyloid PET and hippocampal volume and demographic variables.

Variables in the equation								
							95.0% CI for Exp(B)	
	*B*	SE	Wald	df	Sig.	Exp(B)	Lower	Upper
Zscore(HPCVOL)	−0.617	0.273	5.118	1	0.024	0.540	0.316	0.921
InitialMMSE	−0.651	0.177	13.585	1	<0.001	0.522	0.369	0.737
age	−0.038	0.032	1.391	1	0.238	0.963	0.904	1.025
education	0.020	0.077	0.070	1	0.792	1.020	0.878	1.186
RDAmyloidRead	−2.068	0.751	7.582	1	0.006	0.126	0.029	0.551
Hispanic	1.110	0.559	3.950	1	0.047	3.036	1.015	9.075
sex	0.998	0.521	3.661	1	0.056	2.712	0.976	7.536
LASSI B2 cued recall	−0.350	0.119	8.629	1	0.003	0.705	0.558	0.890
LASSI B2 cued intrusions	0.275	0.086	10.196	1	0.001	1.316	1.112	1.558

In addition, those with higher MMSE scores evidenced a 48% reduction in risk [*B* = 0.651; (SE = 1.77); Wald = 10.94; *p* < 0.001].

As depicted in Table 3, adjusting for all other covariates in the model, a reduced number of correct responses on Cued B2 recall (which taps frPSI) was associated with a 29.5% greater risk for progression to dementia [*B* = -0.350 (SE = 0.19; Wald = 8.63); *p* < 0.003] while semantic intrusions associated with Cued B2 recall was independently associated with a 31.6% greater risk when adjusting for other covariates in the model [*B* = 0.275 (SE = 0.09; Wald = 10.20); *p* = 0.001].

Further, lower hippocampal volume was associated with a faster rate of progression to dementia whereas amyloid-positive individuals evidenced a slower rate of progression to dementia when accounting for other covariates in the model.

It was hypothesized that simultaneous adjustment for other covariates related to amyloid positivity such as hippocampal atrophy, frPSI semantic intrusion errors and lower MMSE scores likely contributed to this result. In fact, post-hoc analyses indicated that when amyloid positivity as a single predictor was considered, it was indeed related to a 2.8 times greater risk of more rapid rate of decline to dementia [*B* = 1,07 (SE.41); Wald = 6,27 *p* < 0.001]. Thus, while underlying amyloid positivity may be a risk factor, it may operate via downstream effects on neurodegeneration and specific cognitive deficits that are greater drivers of rate of progression to dementia.

## 4 Discussion

The current study represents the first attempt to determine how different features of failure to recover from proactive semantic interference (frPSI) are independent predictors of the rate of progression of aMCI to dementia. All aMCI participants were comprehensively evaluated longitudinally as participants of the 1Florida Alzheimer’s Disease Research Center. Simultaneous entry into Cox Proportional Hazards Modeling ensured that we could examine the effects of different aspects of frPSI while appropriately statistically adjusting for the influence of age, education, sex and Hispanic ethnicity. Global mental status for our aMCI participants as well as biomarkers such as amyloid positivity on PET and hippocampal volumes on MRI were also considered.

As expected, higher hippocampal volumes and higher global MMSE scores were predictive of a slower rate of aMCI to dementia. After adjustment for these and other variables in the model, the failure to recover from the effects of PSI (frPSI) as measured by reduced correct responses and number semantic intrusions errors were unique predictors of cognitive decline (although both indices were derived on the same LASSI-L instrument). Thus, frPSI as reflected by Cued B2 performance on the LASSI-L (reduced total correct responses) was independently associated with a 29.5% more rapid rate of progression to dementia, while failures of semantic inhibitory control (i.e., number of semantic intrusion errors) was independently associated with a 31.6% greater rate of progression to dementia regardless of other variables in the model. This supports the notion posited by Cid and Loewenstein (2022) that these are two distinct features of frPSI on the LASSI-L that likely reflect different cognitive mechanisms. Indeed, correlation between Cued B2 Recall and Cued B2 intrusions are (*r* = −0.311) *p* = 0.003 with a modest size (*R*^2^ = 0.0967). The failure to correctly recall List B targets even after another exposure to the words and a second attempt to recall the competing words using cues likely represents a persistent semantic inhibitory deficit due to the influence of a previously encoded semantically related word list (List A). On the other hand, semantic intrusions that occurred despite repeated learning trials of List B, appear to represent more executive disturbances such as deficient self-monitoring, and response inhibition (Curiel et al., 2018b; Loewenstein et al., 2018a; Torres et al., 2019; Cid et al., 2020). Our results support the notion that executive measures such as time to completion on Trails B2 was associated with frPSI SIEs but not frPSI correct responses. I could be argued however, that Trails B taps other cognitive constructs other than executive function, so the use of more executive based measures is warranted in future studies.

The independent predictive utility of different frPSI measures after statistically adjusting for a number of pertinent variables supports this notion that a different process is occurring than with Cued B2 recall (which is highly corelated with other neuropsychological tests of memory). The intrusion errors that occur when an examinee experiences frPSI (Cued B2) is a phenomenon that has previously shown a disconnection between corticolimbic structures such as the medial temporal lobes and prefrontal cortex as measured by resting fMRI (Sánchez et al., 2017). Although beyond the scope of this study, this connectivity among brain structures when assessing these two distinctive subcomponents of frPSI can likely best be determined using functional PET and fMRI studies (Curiel Cid et al., 2023).

Among patients with aMCI, tests utilizing frPSI and semantic intrusions have been shown to better discriminate between those who are amyloid positive versus amyloid negative than the number of correct responses on frPSI (Curiel et al., 2018b; Loewenstein et al., 2018b; Kitaigorodsky et al., 2021; Zheng et al., 2021). On the other hand, among cognitively unimpaired older adults, the number of correct responses on frPSI have been shown to be very sensitive predictors of progression to PreMCI and aMCI independent of etiology (Curiel et al., 2018a; Crocco et al., 2021). In the present longitudinal study, both of these processes had predictive properties with an increased likelihood of progression to dementia. While we adjusted for lower MMSE scores and lower hippocampal volumes which were related to a higher rate of progression to dementia, the finding that amyloid-negative aMCI participants progressed to dementia at a more rapid rate than amyloid-positive aMCI was unexpected. One possibility in our sample is that once the amnestic MCI stage was reached, other, more rapidly progressing neurological conditions other than AD (e.g., FTD, DLBD) and/or vascular contributions known to accelerate dementia may have contributed to this faster decline. Another potential explanation is that simultaneously adjusting for LASSI-L frPSI and amyloid PET measures (which have been shown previously to be strongly related), as well as adjustment demographic factors such as lower MMSE scores and hippocampal atrophy may have distorted the influence of amyloid status. This hypothesis is supported by the data presented in the results section, that amyloid positivity was predictive of rate of cognitive decline when entered in univariate analyses but failed to reach statistically significant when hippocampal volume entered into the adjusted model and was actually a negative predictor when cognitive measures were adjusted in models where all covariates were statistically adjusted for.

The present findings did not suggest predictive effects for sex or educational attainment. However, using the Cox multivariate hazards model, Hispanic individuals demonstrated more rapid decline to dementia. In future studies, larger sample sizes will be required to confirm that this is indeed the case, as our sample of aMCI participants numbered less than 100. Even though our sample was followed for an average of over 26 months (and as long as 55 months) it is acknowledged that it will be important to continue to enroll aMCI participants in our cohort and plan on even longer follow-up periods. A final limitation is that we only included individuals who were classified as aMCI. Additional follow-up periods will allow us to conduct growth curve modeling to examine trajectories of decline across the whole spectrum of patients, including PreMCI (impaired not MCI) and non-amnestic MCI participants. Taken together, the findings regarding the independent associations between different manifestations frPSI as independent predictors of progression to dementia suggest that the failure to recover from proactive semantic interference, despite repeated learning, may have different biological substrates that may be associated with the rate of progression from aMCI to dementia. This is an area clearly worthy of further investigation.

## Data availability statement

The raw data supporting the conclusions of this article will be made available by the authors, without undue reservation.

## Ethics statement

The studies involving humans were approved by This study is an IRB-approved investigation at the University of Miami Miller School of Medicine and Mt. Sinai Medical Center. All procedures performed met all national and international standards for the protection of human subjects. The studies were conducted in accordance with the local legislation and institutional requirements. The participants provided their written informed consent to participate in this study.

## Author contributions

RC: Conceptualization, Investigation, Writing – original draft. EC: Writing – review & editing. RD: Writing – review & editing. DV: Writing – review & editing. BA: Writing – review & editing. MJA: Writing – review & editing. MA: Writing – review & editing. MG: Writing – review & editing. MM: Writing – review & editing. WW: Writing – review & editing. MR: Writing – review & editing. WB: Writing – review & editing. AO: Writing – review & editing. DH: Writing – review & editing. LG: Writing – review & editing. FA: Writing – review & editing. SD: Writing – review & editing. GS: Writing – original draft, Writing – review & editing. DL: Conceptualization, Formal analysis, Writing – original draft.

## Footnotes

^1^http://surfer.nmr.mgh.harvard.edu
